# OnabotulinumtoxinA for Hemicrania Continua: open label experience in 9 patients

**DOI:** 10.1186/s10194-015-0502-z

**Published:** 2015-03-05

**Authors:** Sarah Miller, Fernando Correia, Susie Lagrata, Manjit S Matharu

**Affiliations:** Headache Group, Institute of Neurology and The National Hospital for Neurology and Neurosurgery Queen Square, London, WC1N 3BG UK; Department of Neurology, Centro Hospitalar do Porto, Oporto, Portugal

**Keywords:** Botulinum toxin-A, Hemicrania continua, Treatment, Indomethacin

## Abstract

**Background:**

Hemicrania continua is a strictly unilateral, continuous headache, typically mild to moderate in severity, with severe exacerbations commonly accompanied by cranial autonomic features and migrainous symptoms. It is exquisitely responsive to Indomethacin. However, some patients cannot tolerate treatment, often due to gastrointestinal side effects. Therapeutic alternatives are limited and controlled evidence lacking.

**Methods:**

We present our experience of nine patients treated with OnabotulinumtoxinA for hemicrania continua. All patients were injected using the PREEMPT (Phase 3 REsearch Evaluating Migraine Prophylaxis Therapy) protocol for migraine.

**Results:**

Five of nine patients demonstrated a 50% or more reduction in moderate to severe headache days with OnabotulinumtoxinA with a median reduction in moderate to severe headache days of 80%. Patient estimate of response was 80% or more in five subjects. The median and mean duration of response in the five responders was 11 and 12 weeks (range 6–20 weeks). Improvements were also seen in headache-associated disability

**Conclusions:**

OnabotulinumtoxinA adds a potential option to the limited therapeutic alternatives available in hemicrania continua.

## Background

Hemicrania continua (HC) is a strictly unilateral, continuous headache that is exquisitely responsive to Indomethacin [1]. It is more prevalent in women and usually begins in adulthood [2,3]. The pain is typically of mild to moderate intensity and often involves the forehead, temporal, orbital and occipital regions [3]. Exacerbations of pain are seen in the majority and are commonly accompanied by cranial autonomic features and migrainous symptoms [2,3].

Hemicrania continua is, by definition, exquisitely responsive to Indomethacin [1]. Despite the efficacy of Indomethacin in HC, more than 30% of patients experience adverse effects and 20% have to discontinue the drug [4]. Finding possible therapeutic alternatives to Indomethacin is, thus, of great clinical relevance.

Several other drugs have been reported to be at least partially effective in open-label reports including: cyclooxygenase-2 inhibitors, aspirin, ibuprofen, naproxen, topiramate, melatonin, valproic acid, gabapentin, verapamil and methylprednisolone. Other options are greater occipital nerve blocks (GONB) and neuromodulation. However, none appear to be as effective as Indomethacin.

Even though the exact mechanism of action of OnabotulinumtoxinA (BoNT-A) remains unclear, it is thought to involve multiple mechanisms. Theories include inhibition of neurotransmitter release from motor and sensory nociceptive neurons resulting in interruption of the inflammatory loop promoting peripheral and central sensitization or direct inhibition of central sensitization in the CNS, via axonal transport [5].

The efficacy of BoNT-A in chronic migraine prophylaxis is now well established [6]. Experience in trigeminal autonomic cephalalgias (TACs) is scarce; experience in 14 cluster headache and one Short-lasting Unilateral Neuralgiform Headache with Conjunctival Injection and Tearing (SUNCT) patients have been published [7-11]. In HC, there are two single subject case reports on the use of BoNT-A [12,13]. In the first case, painless autonomic attacks continued, whereas in the second case, autonomic features fully resolved. In this open label study we examine the outcome of nine patients undergoing BoNT-A treatment for HC.

## Methods

Patients receiving BoNT-A with the Headache Group at the National Hospital for Neurology and Neurosurgery were analyzed. Patients were diagnosed with HC in accordance to International Classification of Headache Disorder criteria (ICHD-3beta) [1]. All had unilateral headaches that had responded fully to an indomethacin trial (oral or intramuscular trials, detailed in Table 1). All patients were injected with BoNT-A as per the Phase 3 REsearch Evaluating Migraine Prophylaxis Therapy (PREEMPT) regime for chronic migraine, with patients having a modified regime (exclusion of occipital and cervical paraspinal sites) if they had an occipital nerve stimulator (ONS) in situ [6].

**Table 1:** **Demographic details of patients and treatment**

	**Sex**	**Age at treatment (years)**	**Duration of HC at treatment (years)**	**Phenotype of HC**			**Indomethacin dose required to suppress HC**	**Co-existent headache**	**Previous number of treatments trialled**	**Reasons for administering BoNT-A**	**No of sessions of BoNT-A treatments**	**Average units injected**
				**Location**	**Autonomic symptoms** ^*^	**Migrainous symptoms** ^**Ŧ**^						
**1**	M	19	1	Right	Yes	Nil	225 mg daily	EMWOA (Bilateral)	4	Worsening EM on Indometacin	4	168
**2**	F	61	1	Left	Yes	Yes Visual Aura	150 mg daily	Nil	4	GI-upset	3	165
**3**	M	59	12	Right	Yes	Nil	IM Indometacin test**	Nil	13	Unable tolerate Indometacin; Refractory to other treatments; ONS in-situ	2	175
**4**	M	48	2	Right	Yes	Yes Visual Aura (occasional)	150 mg daily	EMWA (bilateral, once month)	9	GI-upset; peptic ulcer disease; refractory to other treatments - awaiting ONS	2	165
**5**	F	47	9	Right	Yes	Yes	225 mg daily	Past EMWOA (stopped 2004)	3	GI-upset	5	167
**6**	F	49	34	Right	Yes	Yes	150 mg daily	EMWA (bilateral, once month)	6	GI-upset; refractory to other treatments; ONS in-situ	2	110
**7**	F	48	18	Left	Yes	Yes	IM Indometacin test**	ISH	13	GI-upset	2	155
**8**	F	41	8	Right	Yes	No Visual Aura	150 mg daily	EMWOA (side variable/bilateral)	7	GI-upset; wheeze; dizziness; worsening EM	6	168
**9**	F	54	4	Right	Yes	Yes	225 mg daily	Nil	9	GI-upset	2	185
**Mean**		**47**	**10**						**8**		**3**	**162**
**Median (Range)**		**48 (19–61)**	**8 (1–34)**						**7 (3-13)**		**2 (2-6)**	**167 (110–185)**

M, Male; F, Female; HC, Hemicrania continua; EMWA, Episodic migraine with aura; EMWOA, Episodic migraine without aura ISH, Idiopathic stabbing headache; BoNT-A, OnabotulinumtoxinA; GI, Gastrointenstinal; ONS, Occipital nerve stimulator; IQR, Inter-quartile range; ^*^Autonomic symptoms including ptosis, lacrimation, conjunctival injection, meiosis, nasal blockage, rhinorrhea, facial redness, facial sweating, eyelid oedema, restlessness; ^Ŧ^Migrainous symptoms including nausea, vomiting, photophobia, phonophobia, osmophobia, motion sensitivity; **IM Indometacin test blinded placebo test of 100 mg IM Indometacin v normal saline.

All data was collected prospectively with the use of headache diaries. Average monthly scores were calculated from a month pre-treatment and a three-month post-final treatment diary. Headache days were recorded as any day on which the subject recorded HC pain. Subjects were asked to score pain intensity on two scales: 1) pain free, mild, moderate and severe; and 2) verbal rating scale (VRS). Headache load (HAL) was calculated from diaries using the formula: ∑ (severity (VRS) × pain duration (hours). Disability scores consisting of Headache Impact Test (HIT-6), Migraine Disability Assessment (MIDAS) and Hospital Anxiety and Depression scale (HAD) were collected before and after treatment.

Responders to treatment were classified as those achieving a 50% or greater improvement in headache days rated as moderate to severe. Other outcomes included those achieving a 30% and 50% or greater improvement in HAL and 30% or more improvement in headache days rated as moderate to severe.

Median values pre- and post-treatment were compared using Wilcoxon Signed Ranks tests and a statistically significant result set at the 95% level (p = 0.05). Data was processed using IBM SPSS Version 22 for Windows.

The study was approved by Northwick Park Hospital Research Ethics Committee, Hampstead, London, and written consent obtained from all patients.

## Results

A total of nine patients with HC received treatment with BoNT-A, of whom six were females and three males (see Table 1). Median age at time of treatment was 48 years (19–61 years) and median duration of HC was 8 years (1–34 years). During exacerbations, migrainous features were present in six and autonomic features in all subjects. Three patients reported visual auras during exacerbations. Four patients had concomitant episodic migraine (EM) and one co-existent idiopathic stabbing headache. All patients were able to differentiate their co-existent headaches from HC and none of the co-existent headaches had responded to indomethacin trials.

Subjects had failed to respond to a median of seven previous treatments for HC. Two subjects had failed to respond to ONS and one was awaiting ONS implantation.

Reasons for treatment with BoNT-A are summarized in Table 1. Eight patients could not tolerate therapeutic doses of indomethacin due to gastro-intestinal (GI) side effects. Two patients complained of worsening of their EM with indomethacin doses required to suppress HC. Patients had a median of two treatments (range 2–6) with a median BoNT-A dose of 167 units (range 110–185 units) injected at each treatment.

The results of BoNT-A treatment are summarized in Table 2. Five subjects demonstrated a response of 50% or more in reduction of moderate or severe headache days to mild headache days or pain free and were classified as responders to treatment. Six subjects reported a 30% or greater response in reduction of moderate or severe headache days to mild headache days or pain free. The median reduction in total headache days was 90% (range 0–100) (p = 0.026) and in moderate to severe headache days 80% (range 0–100) (p = 0.012). Headache load showed a median improvement of 62% (range 0–100) with six patients demonstrating a 30% and 50% or more improvement. Significant improvements were also seen in average headache hours and average VRS (Table 2). The median subjective duration of response in the five responders post treatment was 11 weeks (range 6–20 weeks, mean 12 weeks). Five subjects reported a subjective benefit of 80% or more in their HC.

**Table 2:** **Headache scores pre- and post- treatment with OnabotulinumtoxinA**

**ID**	**Average headache days/month**			Average moderate -severe days/month*			**Average daily headache hours**			**Average daily VRS**			**Change in headache load (%)**	**Subjective estimate of response**	**Estimated duration of response (weeks)**
	**Pre**	**Post**	**Change %**	**Pre**	**Post**	**Change %**	**Pre**	**Post**	**Change %**	**Pre**	**Post**	**Change %**			
**1**	30	0	100	20	0	100	24	0	100	5	0	100	100	>90%	16
**2**	30	0	100	15	0	100	24	0	100	5	0	100	100	>90%	20
**3**	30	30	0	30	19	37	24	24	0	8	6	25	20	30-50%	5
**4**	30	30	0	30	30	0	24	24	0	7	6	14	0	0	0
**5**	30	3	90	30	3	90	24	6	75	7	10	0	98	80-90%	12
**6**	30	30	0	30	23	23	15	16	0	7	6	14	0	15-25%	4
**7**	30	22	27	21	13	27	24	7	71	7	4	43	55	40%	6
**8**	30	2	93	30	0	100	24	8	67	9	2	78	99	80-90%	6
**9**	30	0	100	30	0	100	24	0	100	8	0	100	100	>90%	9
**Mean**	**30**	**13**	**57**	**25**	**10**	**64**	**23**	**9**	**57**	**7**	**4**	**51**	**62**		**8**
**Median (Range)**	**30 (30)**	**3 (0–30)**	**90 (0–100)**	**30 (15–30)**	**3 (0–30)**	**80 (0–100)**	**24 (15–24)**	**7 (0–24)**	**71 (0–100)**	**7 (5-9)**	**4 (0–10)**	**43 (0–100)**	**98 (0–100)**		**6 (0–20)**

*Response defined as 50% or more improvement in average moderate-severe headache days/month; VRS, Verbal Rating Scale; Pre, Pre-treatment; Post, Post-final treatment.

Four subjects were taking indomethacin prior to BoNT-A and all were able to stop regular use after treatment with two using indomethacin as required at a frequency of less than three times a month.

Headache disability scores showed a trend to improvement after BoNT-A (Table 3). HIT-6 showed a median change of 12 points (p = 0.069). This is above the three-point change suggestive of minimal clinical difference. MIDAS improved by a median of 51 points (p = 0.063).

**Table 3:** **Headache-associated disability scores pre- and post- treatment with OnabotulinumtoxinA**

**ID**	**HIT-6**			**MIDAS**			**HAD-A**			**HAD-D**		
	**Pre**	**Post**	**Change in score**	**Pre**	**Post**	**Change in score**	**Pre**	**Post**	**Change in score**	**Pre**	**Post**	**Change in score**
**1**	65	58	7	73	0	73	10	8	2	9	3	6
**2**	57	36	21	24	0	24	1	0	1	0	0	0
**3**	56	68	−12	52	52	0	3	9	−6	2	12	−10
**4**	68	67	1	105	130	−25	12	6	6	10	8	2
**5**	67	44	23	120	0	120	9	2	7	9	1	8
**6**	63	60	3	121	13	108	16	18	−2	15	15	0
**7**	63	54	9	24	13	9	2	3	−1	5	7	−2
**8**	64	62	2	240	4	236	0	0	0	3	0	3
**9**	76	24	52	51	0	51	12	0	12	4	0	4
**Mean**	**64**	**53**	**12**	**90**	**24**	**66**	**7**	**5**	**2**	**6**	**5**	**1**
**Median (Range)**	**64 (56–76)**	**58 (24–68)**	**7 (−12 to 52)**	**73 (24–240)**	**4 (0–130)**	**51 (−25 to 236)**	**9 (0–16)**	**3 (0–18)**	**1 (−6 to 12)**	**5 (0–15)**	**3 (0–15)**	**2 (−10 to 8)**

HIT-6, Headache Impact Test; MIDAS, Migraine Disability Assessment; HAD-A, Hospital Anxiety and Depression scale (Anxiety); HAD-D, Hospital Anxiety and Depression Scale (Depression); Pre, Pre-treatment; Post, Post-final treatment.

Adverse events were reported in three subjects: one eyebrow ptosis, one frontalis over-activity and one transient worsening in headache before improvement was noted. All adverse events were rated as mild by patients and transient in nature.

## Discussion

This series is the largest so far of BoNT-A treatment for HC. Five out of nine patients showed a greater than 50% reduction in moderate or severe headache days to mild headache days or pain free with a median reduction in moderate and severe days of 80%. All four subjects taking daily indomethacin prior to treatment were able to stop regular use. Five patients reporting an 80% or more improvement in their HC and clinically significant improvements were seen in both HIT-6 and MIDAS.

The patient group had tried a median of seven previous preventatives and had suffered from HC for a median of 8 years at the time of BoNT-A treatment. The refractory nature of the group means that it is doubtful that our observations are due to spontaneous remission. Despite four patients reporting co-existing episodic migraine, all were clearly able to differentiate this from their HC. The phenotype of HC was secure in all subjects and all meet ICHD-3beta criteria including a complete response of their side-locked headache to an adequate indometacin trial. Although a number of subjects report migrainous symptoms associated with HC, this is an accepted feature commented on in epidemiological studies and the ICHD-3beta criteria [1,3]. Given that all patients were carefully phenotyped and could clearly differentiate their episodic migraine attacks from hemicrania continua taken together with the sparse evidence for the efficacy of botulinum toxin in episodic migraine, our data are consistent with a change in HC and not the co-existent episodic migraine.

This series is still small, and this must be considered when interpreting the results. Previous studies of BoNT-A have reported a significant placebo response and we cannot eliminate this as a potential confounding factor in our outcomes. However, the relatively high response rates taken together with the consistent efficacy of repeated BoNT-A injections and a mean duration of effect similar to that seen in other reports in TAC as well as chronic migraine suggest that the response to BoNT-A in this series cannot be attributed entirely to the placebo response [10,12,13].

The exact mechanisms by which BoNT-A produces therapeutic benefit remains unclear, but the neurotoxin is likely to function by multiple mechanisms, suppressing events associated with peripheral and central sensitization. Both migraine and TACs are believed to share a common pathophysiology comprising of the activation of the trigeminovascular system and involvement of neuroactive peptides such as calcitonin gene-related peptide (CGRP), vasoactive peptide (VIP) and glutamate [14]. Animal studies have provided evidence of BoNT-A suppressing nociception in peripheral trigeminovascular neurons and also suppressing CGRP and VIP release from these neurons [5] There is also data to support the hypothesis that the toxin may act via central mechanisms with studies showing retrograde axonal transport of active BoNT-A [15,16]. The potential target of BoNT-A in chronic migraine is the direct blockage of trigeminal neurons providing nociception to the head and face. Suppression of neuro-inflammatory mediator release leads to decreased activation of second-order neurons within the trigemino-cervical complex and brainstem. BoNT-A may therefore be assumed to exert its benefit by repressing the neuro-inflammatory mediators responsible for the maintenance of peripheral and central sensitization [17,18]. It is therefore possible that BoNT-A has a wider therapeutic potential than chronic migraine. It is interesting to speculate that the clinical and functional imaging similarities between migraine and HC may mean that BoNT-A has more of an impact in HC than the other TACs which are much more clinically distinct to migraine [19].

## Conclusion

OnabotulinumtoxinA may be a promising alternative to Indomethacin in patients with HC who do not tolerate the drug. Treatment appears to be associated with a significant improvement in moderate to severe headache days and related disability. It may add another potential therapeutic agent for HC to the limited number available. However, further controlled studies are necessary to clarify the efficacy of BoNT-A in HC.
